# Comparative Analysis of the Antitumor Activity of Cis- and Trans-Resveratrol in Human Cancer Cells with Different p53 Status

**DOI:** 10.3390/molecules26185586

**Published:** 2021-09-14

**Authors:** Christian Leischner, Markus Burkard, Anja Michel, Susanne Berchtold, Heike Niessner, Luigi Marongiu, Christian Busch, Jan Frank, Ulrich M. Lauer, Sascha Venturelli

**Affiliations:** 1 Department of Nutritional Biochemistry, Institute of Nutritional Sciences, University of Hohenheim, 70599 Stuttgart, Germany; christian.leischner@uni-hohenheim.de (C.L.); markus.burkard@uni-hohenheim.de (M.B.); anja.michel56@yahoo.de (A.M.); Heike.Niessner@med.uni-tuebingen.de (H.N.); luigi.marongiu@uni-hohenheim.de (L.M.); 2 Department of Food Biofunctionality, Institute of Nutritional Sciences, University of Hohenheim, 70599 Stuttgart, Germany; jan.frank@nutres.de; 3 Department of Internal Medicine VIII, University Hospital Tuebingen, 72076 Tuebingen, Germany; Susanne.berchtold@uni-tuebingen.de (S.B.); ulrich.lauer@uni-tuebingen.de (U.M.L.); 4 Division of Dermatooncology, Department of Dermatology, University of Tuebingen, 72076 Tuebingen, Germany; 5 Dermatologie zum Delfin, 8400 Winterthur, Switzerland; ch_busch@hotmail.com; 6 German Cancer Consortium (DKTK), DKFZ Partner Site, 72076 Tuebingen, Germany; 7 Department of Vegetative and Clinical Physiology, Institute of Physiology, University of Tuebingen, 72074 Tuebingen, Germany

**Keywords:** resveratrol isomers, cis-resveratrol, trans-resveratrol, antiproliferative effects, tumor cells, hepatocellular carcinoma, colon carcinoma, pancreatic carcinoma, renal cell carcinoma, p53

## Abstract

Resveratrol, a natural plant phytoalexin, is produced in response to fungal infection or− UV irradiation. It exists as an isomeric pair with cis- and trans-conformation. Whereas multiple physiological effects of the trans-form, including a pronounced anti-tumoral activity, are nowadays elucidated, much less knowledge exists concerning the cis-isomer. In our work, we analyzed the antiproliferative and cytotoxic properties of cis-resveratrol in four different human tumor entities in direct comparison to trans-resveratrol. We used human cell lines as tumor models for hepatocellular carcinoma (HCC; HepG2, Hep3B), colon carcinoma (HCT-116, HCT-116/p53^(−/−)^), pancreatic carcinoma (Capan-2, MiaPaCa-2), and renal cell carcinoma (A498, SN12C). Increased cytotoxicity in all investigated tumor cells was observed for the trans-isomer. To verify possible effects of the tumor suppressor p53 on resveratrol-induced cell death, we used wild type and p53-deleted or -mutated cell lines for every tested tumor entity. Applying viability and cytotoxicity assays, we demonstrated a differential, dose-dependent sensitivity towards cis- or trans-resveratrol among the respective tumor types.

## 1. Introduction

**Figure 1:**
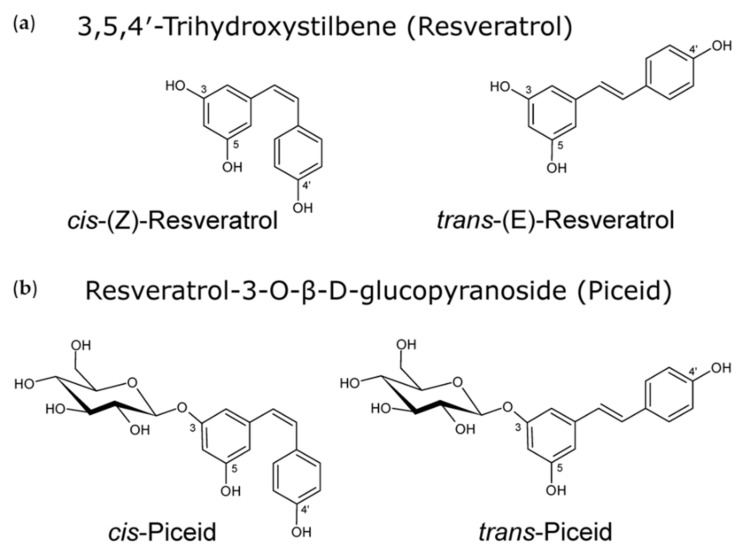
(**a**) Resveratrol is a trihydroxylated stilbene (3,5,4′-Trihydroxystilbene) that exists in two isomeric forms, cis- and trans-. (**b**) In nature, resveratrol occurs mainly as the cis- or trans-glycopyranoside piceid (resveratrol-3-O-β-glycopyranoside). The structural formulas were created with ACD/Chemsketch (Advanced Chemistry Development, Inc.; Toronto, ON, Canada; corresponding to Leischner et al., 2016).

Resveratrol (3,5,4′-trihydroxystilbene) is a trihydroxylated stilbene that belongs to a subclass of plant polyphenols present in many dietary plants such as grapes, peanuts, soy, hop, and berries. Resveratrol exists in two isomeric forms, cis-(Z) and trans-(E) (Figure 1), of which trans-resveratrol has been mainly investigated to date.

The trans-isomer exhibits multiple properties such as antioxidant [1,2], anti-inflammatory [3], neuroprotective [4], immunomodulatory [5], antiproliferative [6,7], and anti-metastatic [8,9] effects. In the context of human hepatoblastoma cells, trans-resveratrol is an inhibitor of classical histone deacetylases of classes I, II, and IV, which are involved in cancer development and progression [10]. Moreover, trans-resveratrol enhances NK cell killing activity by induced expression of activating cell surface receptors such as natural-killer group 2 and member D (NKG2D) [11]. Naturally, the trans-isomer can be converted to the cis-configuration by UV irradiation. Resveratrol and its consumption, primarily in the form of red wine, gained public attention as it was held responsible for the so-called “French paradox”, a term illustrating the lower mortality rate from coronary heart disease in France compared to the rest of Europe and the USA, despite a high intake of saturated fat [12]. It is known that grapes mainly contain trans-resveratrol in its glycosidic form (trans-piceid, trans-resveratrol-3-O-β-D-glucopyranoside; Figure 1), in contrast to red wines, which are a source of both aglycones of cis- and trans-resveratrol in often comparable amounts due to sugar cleavage during vinification [13]. To date, piceid and aglycone content of both resveratrol isomers have been determined for a multitude of wines of different origins [14,15,16], but laboratory investigations still mostly concentrate on the biological effects of trans-resveratrol. The characteristics of the cis-isomer are much less studied, probably due to its lower steric stability compared to the trans-isomer, its extreme photo-sensitivity [17] under standard laboratory conditions, and the fact that it was not commercially available as pure compound until recently. For a long time, cis-resveratrol could only be obtained by photodiastereomerization of the trans-isomer [18]. The natural origin of the cis-isomer can further be the production from trans-resveratrol by yeast isomerases during wine fermentation, the release from resveratrol polymers (viniferins), or from resveratrol glycosides [19,20].

Integrin α_V_β_3_, a cell surface protein bearing a stilbene binding domain, can serve at least as a trans-resveratrol receptor that activates the mitogen-activated protein kinase (MAPK; extracellular signal-regulated kinase (ERK) 1/2) signal transduction cascade [21]. Whether or not the cis-isomer exerts its effects via the same receptor is not known.

Comparisons of antioxidant effects between the isomers show qualitatively similar results in blocking extra- and intracellular reactive oxygen species (ROS) production by inflammatory rat peritoneal macrophages via inhibiting NAD(P)H oxidase activity [22]. In addition, both isomers inhibit inducible nitric oxide synthase (NOS)-2 and inducible cyclooxygenase (COX)-2 protein synthesis, and attenuate prostaglandin E2 (PGE2) production [22,23]. Both isomers have a comparable capability to inhibit protein tyrosine kinase (PTK) and protein kinase C (PKC), thus contributing to anti-tumor activity [24].

Structural analysis of the resveratrol isomers indicates a weaker potency of the (Z)-configuration in proliferation and cytotoxicity assays with the human colon carcinoma cell line SW480, but substitution of the hydroxyl groups by methoxyl groups results in a stronger inhibitory effect than the (E)-configuration of resveratrol itself and after methoxylation [25]. Similarly, methoxylation of trans- and cis-resveratrol has a major effect on the motility of B16F10 melanoma cells, with methoxylation of the cis-isomer likely having a greater impact by reducing β-tubulin protein levels, which is observed exclusively with the cis-polymethoxy derivative [26]. Further, novel resveratrol derivatives were found to disrupt the tubulin network in human cancer cell lines (HepG2, A549, and Hela) [27]. In addition, various cis-stilbene compounds have a higher growth inhibiting potency compared with their corresponding trans-isomers towards three isogenic cell lines derived from colorectal carcinoma HCT-116 cells, with 3,4,5,4′-tetramethoxy-cis-stilbene having the lowest IC_50_ of 20 nM [28], indicating the importance of the styrene double-bond orientation. Interestingly, cis- and trans-resveratrol execute opposite effects on tyrosyl-tRNA synthetase-regulated poly-ADP-ribose polymerase (PARP)1 activation. In this context, cis-resveratrol induces a protective stress response that is inhibited by trans-resveratrol [29]. Trans-resveratrol shows better encapsulation efficacy in β-casein micelles compared to cis-resveratrol [30].

In order to further evaluate cis-resveratrol, which has comparatively been little investigated in the tumor context, the present study aimed to systematically compare the antiproliferative efficacy of the two resveratrol isomers towards four common tumor entities. In addition, the influence of p53 status was investigated in order to possibly identify patients who might particularly benefit from the administration of one or even both resveratrol isomers in future clinical trials (Table 1).

## 2. Results

Cytotoxic activity and anti-proliferative effects of trans- versus cis-resveratrol.

### 2.1. Western Blot and CellTiter-Blue^®^ Assay

To initially evaluate the effect of resveratrol on p53 expression, Western blotting was performed using HepG2 and HCT-116 cells. No changes were detectable upon exposure to 10 µM cis- or trans-resveratrol (Figure S1).

The fluorometric CellTiter-Blue^®^ (CTB) cell viability assay provides information on the number of viable cells by measuring the metabolic capacity of cells to reduce the given dye resazurin to its fluorescent product resorufin. Dead cells provide no signal and impaired cells show lower turnover rates.

**Figure 2:**
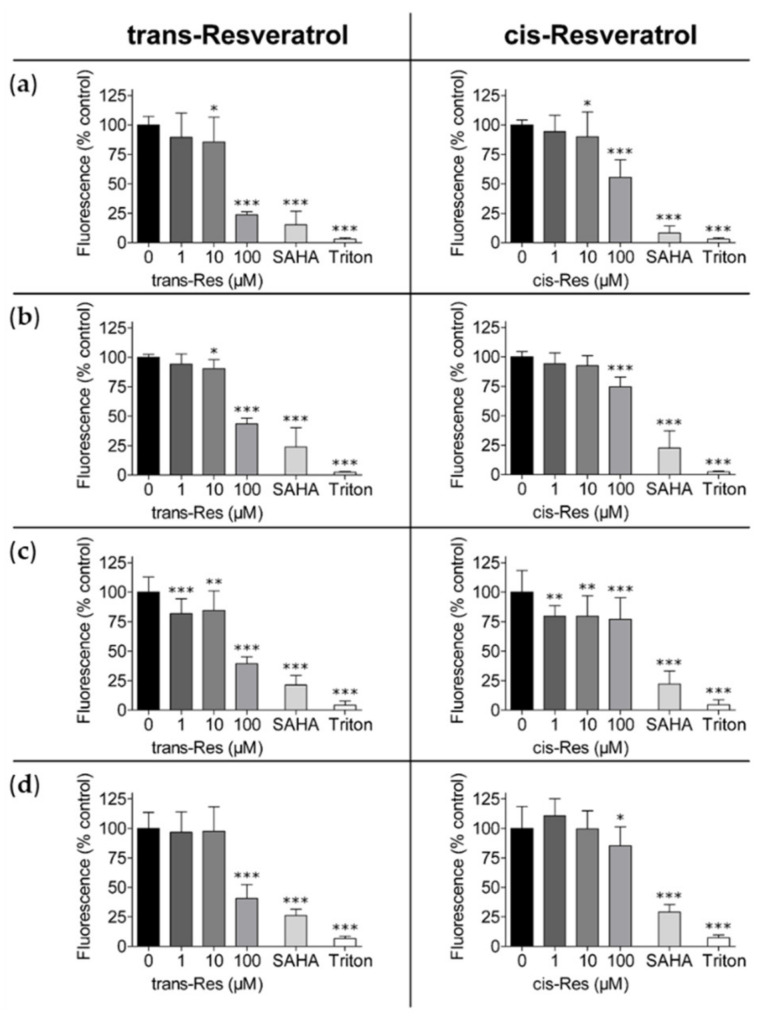
Reduced viability of hepatoma and colon carcinoma cells with different p53 status by trans- and cis-resveratrol shown by CTB assay. (**a**) HepG2, (**b**) Hep3B (hepatoma), (**c**) HCT-116, and (**d**) HCT-116/p53^(−/−)^ (colon carcinoma) cells were exposed to different concentrations of trans- and cis-resveratrol (1 µM, 10 µM, 100 µM) for 72 h; 10 µM SAHA served as positive control and 1% (*v*/*v*) Triton X-100 was added as a cell death control. The experiments were replicated in three independent experiments, each performed in pentaplicates. Results are shown as percentages compared to the untreated control. Error bars represent mean ± SD, statistical analysis with the Dunnet’s multiple comparison test, confidence interval 95%. *: *p* ≤ 0.05; **: *p* ≤ 0.01; ***: *p* ≤ 0.001. CTB, CellTiter Blue^®^; Res, resveratrol; SAHA, suberoylanilide hydroxamic acid.

CTB assay measurements have always shown that trans-resveratrol was the more potent isomer. The results for the hepatoma cell lines HepG2 and Hep3B, and the colon carcinoma cell lines HCT 116 and HCT-116/p53^(−/−)^ are shown in Figure 2. Here, trans-resveratrol at a concentration of 100 µM reduced viability in a range between 75% (in HepG2 and MiaPaCa-2) and 50% (in Capan-2) compared to untreated controls. In comparison, the cis-isomer reduced viability in a range between 50% (in HepG2) and 20% (in SN12C), relative to untreated controls (Figure 2 and Figure S2). In addition, the renal carcinoma cell line SN12C and the pancreatic cancer cell line Capan-2 had a stronger growth inhibition after exposure to 100 µM trans-resveratrol when compared to 10 µM suberoylanilide hydroxamic acid (SAHA), which was barely effective in Capan-2 cells (Figure S2). HepG2, Hep3B, and A498 cells were slightly sensitive to trans-resveratrol at lower concentration ranges, while, for the remaining cell lines, only 100 µM trans-resveratrol was effective (Figure 2 and Figure S2). Trans-resveratrol always had about twice the inhibitory activity compared to the cis-isomer at 100 µM; only Capan-2 cells displayed a similar susceptibility to both resveratrol-isomers.

### 2.2. Sulforhodamine B and Lactate Dehydrogenase Assays

**Figure 3:**
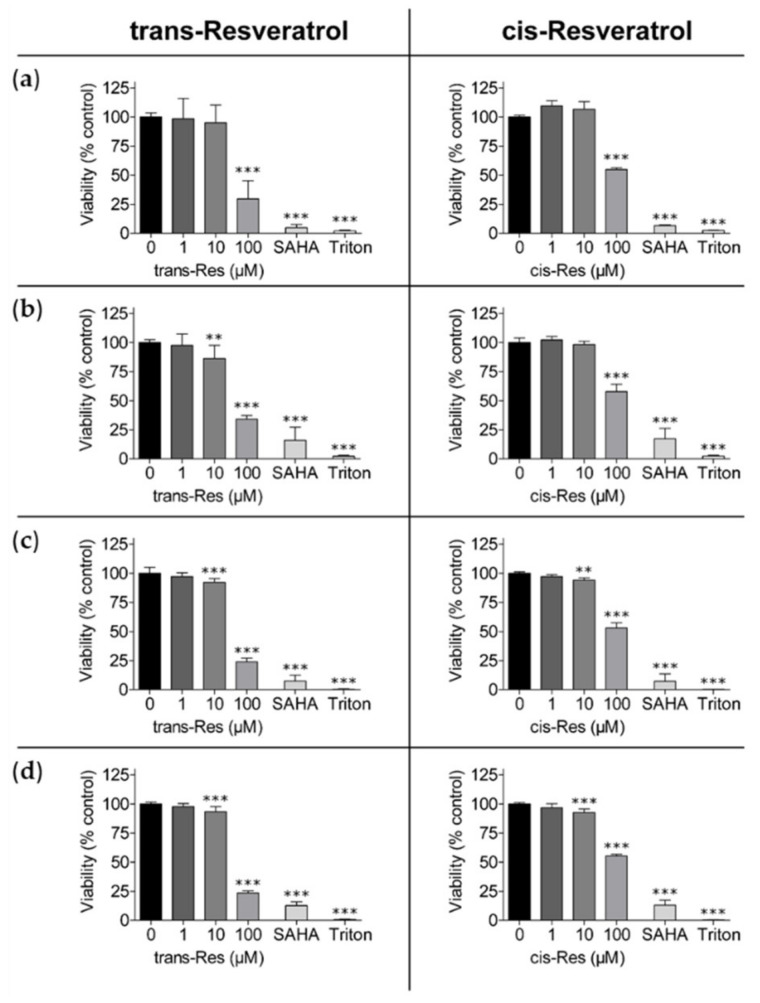
Reduced cell mass of hepatoma and colon carcinoma cells with different p53 status by trans- and cis-resveratrol shown by SRB assay. (**a**) HepG2, (**b**) Hep3B (hepatoma), (**c**) HCT-116, and (**d**) HCT-116/p53^(−/−)^ (colon carcinoma) cells were exposed to different concentrations of trans- and cis-resveratrol (1 µM, 10 µM, 100 µM) for 72 h; 10 µM SAHA served as positive control and 1% (*v*/*v*) Triton X-100 was added as a cell death control. The experiments were replicated in three independent experiments, each performed in triplicates. Results are shown as percentages compared to the untreated control. Error bars represent mean ± SD, statistical analysis with the Dunnet’s multiple comparison test, confidence interval 95%. **: *p* ≤ 0.01; ***: *p* ≤ 0.001. Res, resveratrol; SAHA, suberoylanilide hydroxamic acid; SRB, sulforhodamine B.

By using the colorimetric sulforhodamine B (SRB) assay it is possible to differentiate the cell mass as surrogate for cell proliferation by the nonspecific staining of cell proteins [38]. The cytotoxic potential of the resveratrol isomers at increasing concentrations was inferred from their antiproliferative activity, described above. For each cell line, the trans-isomer of resveratrol always had a more pronounced antiproliferative activity (Figure 3 and Figure S3). Only the Capan-2 cell line was equally sensitive to both isomeric forms of resveratrol. In all other cell lines, the inhibitory efficiency of 100 µM trans-resveratrol was about twice that of 100 µM cis-resveratrol, and even higher in the MiaPaCa-2 and SN12C cell lines (Figure 3 and Figure S3). Weak anti-proliferative effects were detectable in most cell lines above 10 µM trans-resveratrol, except for HepG2 and MiaPaCa-2. For cis-resveratrol, an anti-proliferative effect above 10 µM was detectable only in SN12C and HTC-116 (p53-wt and p53-deleted) cells. No suppressed proliferation was observed at lower compound concentrations. Interestingly, 10 µM SAHA, displaying the most pronounced antiproliferative effects towards most cell lines, induced growth suppression in Capan-2 cells comparable to 100 µM cis-/trans-resveratrol. Moreover, reduction of cell proliferation of the renal carcinoma cell lines A498 and SN12C was even less susceptible towards SAHA when compared to 100 µM trans-resveratrol.

In addition to the SRB assay data, we obtained additional cytotoxic parameters by performing a lactate dehydrogenase (LDH) assay with the supernatant of the cellular experiments. Cells with an impaired or damaged cell membrane release LDH into the cell culture medium, which remains stable and whose kinetic activity can be measured by converting pyruvate to lactate via the simultaneous oxidation of NADH to NAD^+^.

This assay only detects extracellular LDH activity in the supernatant and no intracellular activity. For all cell lines, no clear increase in LDH activity in the supernatant could be detected upon exposure to cis- or trans-resveratrol for 72 h; only a slight increase was measurable for 100 µM trans-resveratrol in the case of A498 cells. Exposure to 10 µM SAHA as control resulted in a slightly higher LDH activity, except for the cell line Capan-2, which yielded no difference compared to the untreated control (Figure S4). Lysis of the cells with 1% (*v*/*v*) Triton X-100 revealed variable LDH levels within the different cell lines with maximum values for the colon carcinoma cell lines HCT-116 and HCT-116/p53^(−/−)^, as well as for A498 (renal cell carcinoma) and MiaPaCa-2 (pancreatic carcinoma), and minimal values for Capan-2 (pancreatic carcinoma) and Hep3B (hepatocellular carcinoma). Exposure to resveratrol for 72 h possibly causes a reduction in intracellular LDH activity, which has been described, e.g., in HCT-116 and Caco-2 cells (colon carcinoma) [39].

The SRB assay registers detached cells with impaired viability, together with dead cells corresponding to lower optical density values, whereas the LDH assay determines only the externalized LDH activity of cells with damaged membranes. Our results have shown that the SRB assay was more suitable in the context of this work for measuring cytotoxicity compared with the LDH assay. The same was true for the CTB assay, which provided better insight into cytotoxicity.

**Figure 4:**
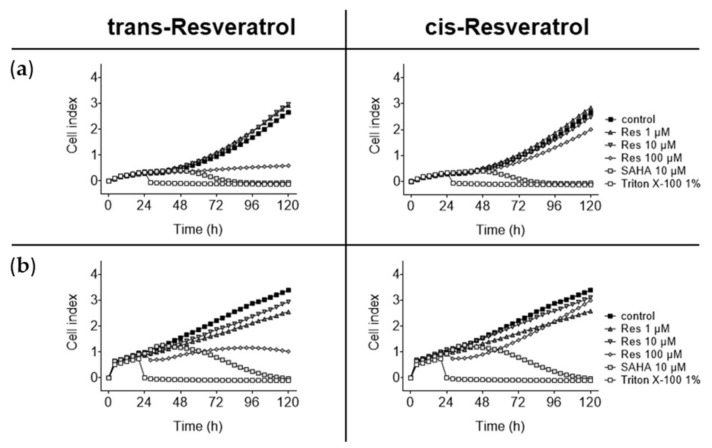
Real time proliferation assay. (**a**) HepG2 and (**b**) Hep3B cells were exposed to different concentrations of trans- and cis-resveratrol (1 µM, 10 µM, 100 µM) or solvent as control and monitored for 96 h by measuring cellular impedance at 30 min intervals. In the graph, 4 h intervals are shown. The xCELLigence^®^ SP system was used for this purpose. Exposure to 10 µM SAHA was used as positive control and exposure to 1% (*v*/*v*) Triton X-100 resulted in complete cell death. Data were calculated using RTCA Software Pro 2.3.2 (Agilent Technologies Inc., Santa Clara, CA, USA). For each cell line, the mean values of one representative run out of three, measured as quadruplicates, are shown. Res, resveratrol; SAHA, suberoylanilide hydroxamic acid.

In order to additionally analyze the temporal onset of growth impairment and cell death, a real time proliferation assay was performed using HepG2 and Hep3B cell lines as examples. In both cases, 100 µM trans-resveratrol induced delayed cell proliferation compared to 100 µM cis-resveratrol (Figure 4). An amount of 100 µM trans-resveratrol directly delayed Hep3B proliferation and stopped cell growth after approximately 72 h; in the case of HepG2, no proliferation was detectable immediately after the addition of 100 µM trans-resveratrol. Hep3B cells had some sensitivity to both resveratrol-isomers at lower concentrations. SAHA induced pronounced effects after only 12 h in Hep3B and after 24 h in HepG2, until most cells were dead after 96 h (Hep3B) and 48 h (HepG2), respectively.

### 2.3. Evaluation of Cell Death (Tetramethylrhodamine Ethyl Ester Staining)

**Figure 5:**
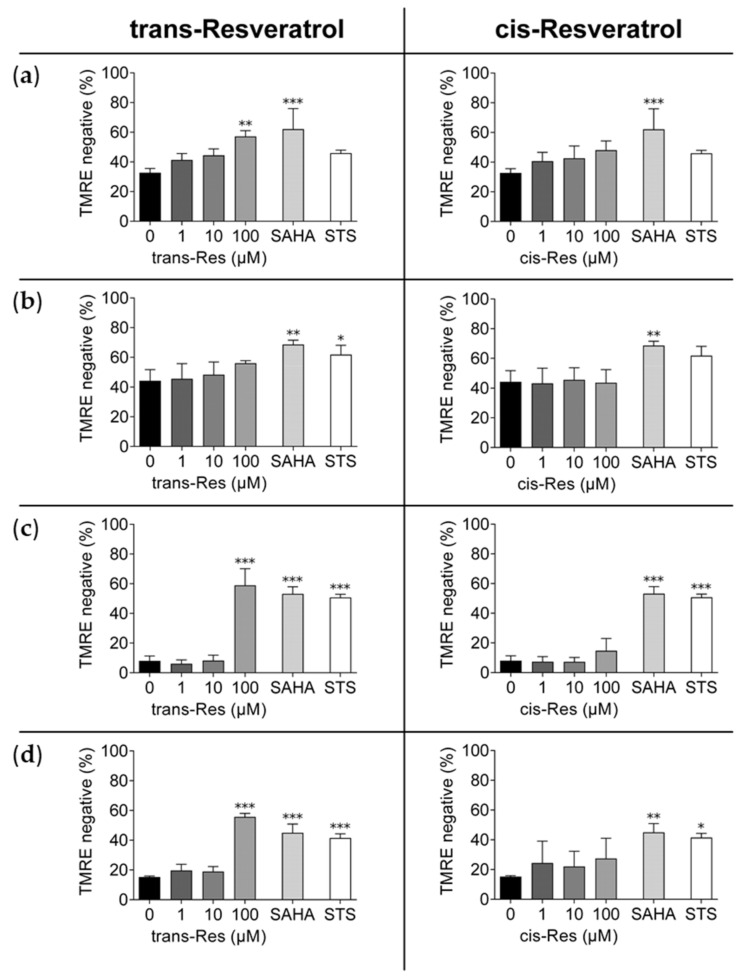
Reduced TMRE staining of mitochondria in hepatoma and colon carcinoma cells by trans- versus cis-resveratrol, evaluated by flow cytometry. (**a**) HepG2, (**b**) Hep3B (hepatoma), (**c**) HCT-116, and (**d**) HCT-116/p53^(−/−)^ (colon carcinoma) cells were exposed to different concentrations of trans- and cis-resveratrol (1 µM, 10 µM, 100 µM) for 72 h; 10 µM SAHA was applied as positive control and 5 µM STS as control for apoptotic cell death. Experiments were replicated in three independent experiments. Error bars represent mean ± SD, statistical analysis with the Dunnet’s multiple comparison test, confidence interval 95%. *: *p* ≤ 0.05; **: *p* ≤ 0.01; ***: *p* ≤ 0.001. Res, resveratrol; SAHA, suberoylanilide hydroxamic acid; STS, staurosporine; TMRE, tetramethylrhodamine ethyl ester.

A decrease of mitochondrial transmembrane potential as a result of leakage of the inner mitochondrial membrane may serve as a sign of apoptosis in progress [40]. Tetramethylrhodamine ethyl ester (TMRE) is a positively charged dye that allows flow cytometric analysis because of its accumulation in active mitochondria [41]. Polarized mitochondria from non-apoptotic cells emit a strong fluorescent signal, whereas apoptotic cells do not. Measurable mitochondrial damage thus correlates with cell death. Exposure of the hepatocellular carcinoma cell line HepG2 to 100 µM cis- and trans-resveratrol resulted in depolarization of the mitochondrial membrane (Figure 5), with the trans-isomer being about 10% more effective. The trans-isomer also showed stronger efficacy in Hep3B and much stronger efficacy in both colon carcinoma cell lines compared to the cis-isomer and induced more cell death in HCT-116 and especially in p53-deleted HCT-116/p53^(−/−)^ cells compared to 10 µM SAHA. Overall, trans-resveratrol induced membrane potential degradation rates of about 60% in the hepatoma and colon carcinoma cells, respectively. Apoptosis-inducing 5 µM staurosporine (STS) triggered cell death in all lines, with no significant effect in HepG2, and 10 µM SAHA was generally more potent than STS (Figure 5). Representative FACS plots are shown in Figure S5.

### 2.4. Cell Cycle Analysis/Evaluation of Apoptosis (Propidium Iodide Staining)

**Figure 6:**
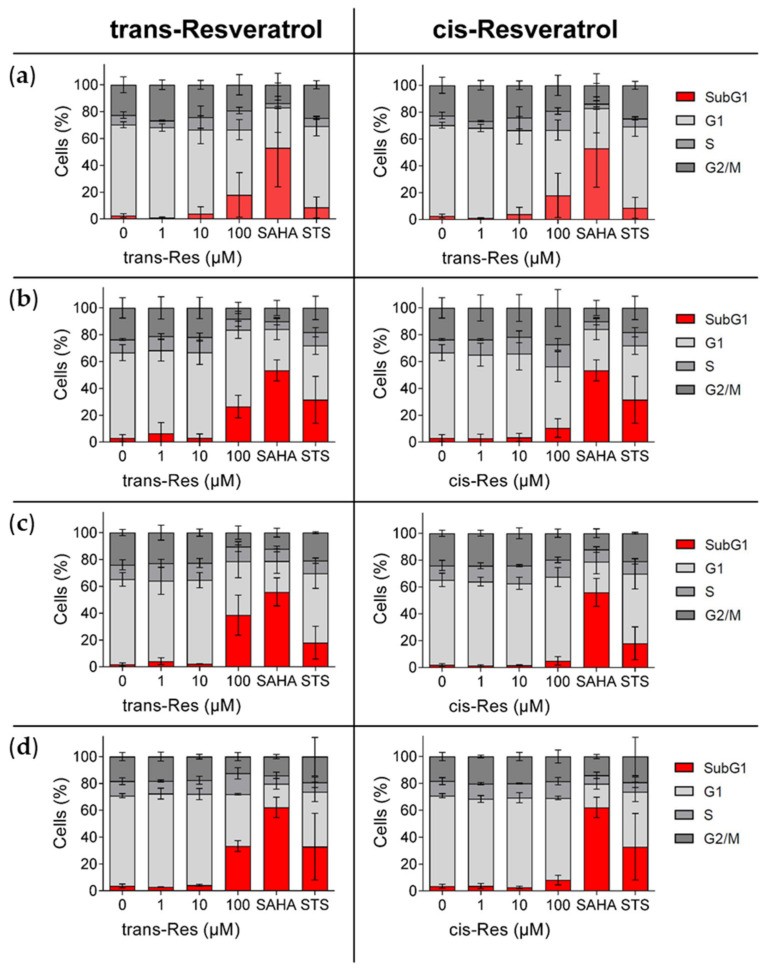
Cell cycle analysis and evaluation of apoptosis in hepatoma and colon carcinoma cells by trans- versus cis-resveratrol, measured after propidium iodide staining by flow cytometry. (**a**) HepG2, (**b**) Hep3B (hepatoma), (**c**) HCT-116, and (**d**) HCT-116/p53^(−/−)^ (colon carcinoma) cells were exposed to different concentrations of trans- and cis-resveratrol (1 µM, 10 µM, 100 µM) for 72 h; 10 µM SAHA was applied as positive control and 5 µM STS as control for apoptotic cell death. The proportion of the subG1 fraction of the cells within the cell cycle phases is shown in red. Experiments were replicated in three independent experiments. Error bars represent mean ± SD. Res, resveratrol; SAHA, suberoylanilide hydroxamic acid; STS, staurosporine.

Staining with propidium iodide (PI) followed by flowcytometric analysis was performed to evaluate whole cell cycle changes that are induced by trans- and cis-resveratrol, respectively (Figure 6). Induction of apoptosis (displayed as SubG1 fraction) was more pronounced for 100 µM trans-resveratrol when compared to cis-resveratrol in all cell lines tested (HepG2, Hep3B, HCT-116, and HCT-116/p53^(−/−)^ cells). To further confirm the results of the cell cycle analysis, the ApoTox-Glo^TM^Triplex assay was performed with the highest used concentrations of trans- and cis-resveratrol (100 µM) (Figure S6).

## 3. Discussion

### Impact of p53 Status on the Anti-Proliferative Potential of Cis- and Trans-Resveratrol

Slightly more than 50% of human cancers harbor mutations in the p53 gene [42]. The transcription factor p53 plays an important role in DNA damage recognition, followed by control of critical cell cycle checkpoints, e.g., by inducing the cyclin-dependent kinase (CDK) inhibitor p21^Waf1/Cip1^, leading to G1 and G2 cell cycle arrest to allow time for DNA repair mechanisms, or it acts later, e.g., in mitochondria-mediated apoptosis, by activating transcription of members of the pro-apoptotic Bcl-2 protein family such as Bax, Noxa [43], or p53AIP1 [44], and finally caspase activation [45]. Among numerous other implications, the expression of apoptosis-mediating cell surface receptors such as Fas/APO1 [46] or Killer/DR5 [47] is induced by activated p53.

Therefore, defined tumor cell lines with different p53 status (functional p53 and non-functional p53) were included in this study. The involvement of p53 in resveratrol-induced apoptosis was previously shown for mouse fibroblasts that were either p53 wild-type (wt) (p53^(+/+)^) or p53-deficient (p53^(−/−)^), with apoptosis only occurring in p53 wt cells [48]. Similarly, for several human breast cancer cell lines with different p53 gene status, resveratrol-induced apoptosis was only observed in cells expressing p53 wt [49]. Other examples of p53-dependent trans-resveratrol-induced apoptosis included prostate cancer [50], glioma [51], and head and neck squamous cell cancer cells [52]. More recently, transient transfection of p53 wt rendered non-small lung cancer cells H1299 with partial deletion of the p53 gene susceptible to the pro-apoptotic effects of trans-resveratrol [53]. However, experiments with tetramethoxylated cis- and trans-stilbene demonstrated p53-independent growth inhibition of p53 wt and p53 null (p53^(−/−)^) isogenic HCT-116 cells [28]. A p53- and p21-independent apoptosis induction by trans-resveratrol was further described in the same cell lines and additionally in HCT-116/p21^(−/−)^ cells [54].

In our in vitro experiments with different tumor cells, SRB viability assays showed no differences in growth inhibition between p53 wt and p53-deficient cells after stimulation with 100 µM trans-resveratrol. As an exception, trans-resveratrol inhibited the pancreatic carcinoma cell line MiaPaCa-2 (p53-mutated) significantly more than the Capan-2 cell line (p53 wt). Capan-2 was the only cell line with a similar degree of proliferation inhibition by trans- and cis-resveratrol, which was confirmed by SRB and CTB viability assays. Cell lines with impaired p53 status (Hep3B, HCT-116/p53^(−/−)^, MiaPaCa-2, SN12C) showed slightly lower sensitivity to cis-resveratrol-mediated cytotoxicity than their wt counterparts (HepG2, HCT-116, Capan-2, A498), resulting in lower growth inhibition according to the SRB results. Flow cytometric analysis after TMRE and PI staining confirmed superior induction of tumor cell death by the trans-isomer in all hepatoma and colon carcinoma cell lines, irrespective of their p53 status, which was additionally confirmed by a commercially available viability assay.

The efficacy of resveratrol isomers in inhibiting cellular proliferation was cell line-dependent, which was also true for the control compound SAHA. Significant effects could only be detected at the highest concentration of 100 µM, which applied to both isomeric forms. Thereby, the trans-isomer always showed higher potency compared to cis-resveratrol in different proliferation and viability assays, with the sole exception of Capan-2 cells, which were equally affected by both isomers.

Our results are in line with other comparative studies concerning the biological effects of cis- versus trans-resveratrol, which demonstrated a slightly lower antiproliferative effect of the cis-isomer on, e.g., the androgen-independent prostate cancer cell line PC-3 in a concentration range from 1 to 100 µM [55]. In addition, cis-resveratrol induced a greater decrease in collagen-induced platelet aggregation [56], but was not as potent as the trans-isomer in a structure-activity study of potential antineoplastic activity of various stilbene derivatives in several cancer cell lines [57].

Interestingly, cis-orientation in stilbene-derivatives substituted with other functional groups, e.g., by methoxylation, showed increased antitumor functionality compared to their trans-orientation counterparts. This suggests a strong role of substituent orientation, yet the exact drug signaling pathway remains to be elucidated. Further, bioavailability should be enhanced by hydroxyl group substitution, because trans-resveratrol alone only reaches a maximum plasma concentration of just under 2 µM, tested with administered ^14^C-labeled trans-resveratrol [58], which is not comparable to the effective treatment concentration of 100 µM in the present study.

Furthermore, resveratrol is rapidly metabolized after ingestion, mainly by sulfate and glucuronic acid conjugation, additionally by hydrogenation of the double-bond, followed by rapid excretion [59]. However, comprehensive pharmacokinetic data from metabolic studies on the bioavailability, transformation, excretion, or achievable plasma levels of cis-resveratrol or its metabolite conjugates are not yet available. For cis-resveratrol to be an effective therapeutic agent against cancer, information in this regard is critical.

Taken together, cis-resveratrol could serve as a lead structure for the development of small molecules with high bioavailability and antitumor activity while maintaining a low cytotoxicity profile without pronounced unwanted side effects. In addition, daily dietary intake of resveratrol-isomers could serve as long-term chemoprevention for hepatocellular cancer, colorectal cancer, and prostate cancer [60,61,62].

## 4. Materials and Methods

### 4.1. Cell Culture and Reagents

Human hepatoma cell lines HepG2 (hepatoblastoma, DSMZ-No: ACC 180) and Hep3B (hepatocellular carcinoma, DSMZ-No: ACC 93), human colon carcinoma cell line HCT-116 (DSMZ-No: ACC 581), and human kidney carcinoma cell line A498 (DSMZ-No: ACC 55) were obtained from the German Collection of Microorganisms and Cell Cultures (Deutsche Sammlung von Mikroorganismen und Zellkulturen (DSMZ), Braunschweig, Germany). Human pancreatic carcinoma cell line Capan-2 (ATCC-No: HTB-80) was purchased from the American Type Culture Collection (ATCC, LGC Standards, Wesel, Germany). MiaPaCa-2 pancreatic carcinoma cell line (ECACC-No: 85062806) was provided by the European Collection of Authenticated Cell Cultures (ECACC, Salisbury, UK). p53-deficient HCT-116 cells were obtained from B. Vogelstein (Johns Hopkins University, Baltimore, MD), which were originally developed by targeted homologous recombination [34]. Disruption of the p53 gene product was additionally verified by Western blotting (Figure S1). Human renal carcinoma cell line SN12C was part of the NCI-60 tumor cell panel obtained from Charles River Laboratories (Charles River Laboratories Inc., Sulzfeld, Germany).

Cell line HepG2 was maintained in Dulbecco´s modified Eagle´s medium, low glucose (DMEM, Sigma–Aldrich, Steinheim, Germany), supplemented with 10% (*v*/*v*) fetal calf serum (FCS; Biochrom, Berlin, Germany) and an additional 1% (*v*/*v*) L-Glutamine (200 mM, Sigma–Aldrich); the other cell lines were cultivated in DMEM, high glucose (Sigma–Aldrich), supplemented with 10% (*v*/*v*) FCS. Pancreatic carcinoma cells Capan-2 were cultivated in McCoy´s 5A modified medium (Biochrom) supplemented with 10% (*v*/*v*) FCS and 1% (*v*/*v*) L-Glutamine (200 mM). All cell lines were stored at 37 °C in a humidified atmosphere containing 5% CO_2_.

Cis- and trans-resveratrol were obtained in solid form from Santa Cruz Biotechnology, Inc. (cis-Resveratrol: CAS 61434-67-1; trans-Resveratrol: CAS 501-36-0, Heidelberg, Germany), solved in dimethyl sulfoxide (DMSO, CAS 67-68-5, Sigma–Aldrich), and kept as 100 mM stock solutions at −20 °C. SAHA was purchased from Cayman (CAS 149647-78-9, Ann Arbor, MI, USA) and likewise kept as 10 mM stock solution solved in DMSO. STS was purchased from Enzo Life Sciences (Loerrach, Germany) and kept as 500 µM stock solution in DMSO at −20 °C. p53 antibody (DO-1): sc-126 from mouse was purchased from Santa Cruz Biotechnology, monoclonal anti-Vinculin antibody from mouse was obtained from Sigma–Aldrich, and goat anti-mouse IgG (H+L)-horseradish peroxidase (HRP) conjugate from Bio-Rad Laboratories (Munich, Germany).

### 4.2. Immunoblotting

Human hepatoma cell lines HepG2 and Hep3B (1 × 10^6^ cells/dish), as well as colon carcinoma cell lines HCT-116 and HCT-116/p53^(−/−)^ (5 × 10^5^ cells/dish), were seeded in 60 mm cell culture dishes (Corning, NY, USA) in a volume of 4 mL and incubated with 10 µM trans- and cis-resveratrol the next day. After an incubation period of 72 h, cells were washed once with PBS, harvested by scraping, and centrifuged. The cell pellet was subsequently resuspended in 100 µL lysis buffer (1% (*v*/*v*) Igepal CA-630 (Sigma–Aldrich), 50 mM Tris base (pH 7.5), 150 mM NaCl, and one protease inhibitor cocktail tablet (cOmplete ULTRA tablets, Mini; Roche) in 10 mL lysis buffer). The lysates were thawed three times and refrozen in liquid nitrogen and finally freed from cell debris by centrifugation for 10 min at 18,000 g. The supernatant was stored at −20 °C for future use. Protein content of the samples was determined by the Bradford protein assay. To prepare lysates, a volume of 20 µL was adjusted to a concentration of 2.5 mg protein/mL, and 4 µL of 6-fold loading buffer (100 mM Tris base (pH 6.8), 20% (*v*/*v*) glycerol, 4% (*w*/*v*) sodium dodecyl sulfate (SDS), 10% (*v*/*v*) 2-mercaptoethanol, 6 mg bromophenol blue in 10 mL buffer volume) was added and heated at 95 °C for 5 min. Lysates were separated on 8% SDS polyacrylamide gels and transferred to polyvinylidene difluoride (PVDF) membranes (Hybond-P, GE Healthcare, Munich, Germany). The membrane portion containing the p53 bands was blocked with Roti Block blocking solution (Roth) for 1 h, followed by overnight incubation with anti-p53 mouse antibody (1:500, Santa Cruz) in Roti Block solution at 4 °C. The portion of the membrane with vinculin bands was blocked overnight at 4 °C in TBST (Tris-buffered saline (TBS)-Tween 20; 10 mM Tris base (pH 7.6), 150 mM NaCl, 0.05% (*v*/*v*) Tween 20) with 5% (*w*/*v*) nonfat dry milk powder (Roth). Incubation of the membranes with anti-vinculin mouse antibody solution (1:5000, Sigma–Aldrich, in TBST) was then performed for 1 h at room temperature. Membranes were then washed three times in TBST, incubated for 45 min with HRP-conjugated anti-mouse antibody (1:8000, Bio-Rad Laboratories) in TBST, and finally washed six times in TBST alone. Detection was performed using ECL Western blotting detection system on Hyperfilm ECL (GE Healthcare).

### 4.3. CellTiter-Blue^®^ Cell Viability Assay

To estimate the effects on cell viability, CellTiter-Blue^®^ Cell Viability Assay from Promega (G8081, Mannheim, Germany) was performed. In 96-well plates 5 × 10^3^ cells of the different cell lines (except 7.5 × 10^3^ cells of HepG2 and Capan-2 due to slower growth rates) were seeded in a volume of 100 µL as pentaplicates, incubated overnight, and after removing the medium, exposed to medium containing 0.1, 1, 10, or 100 µM of cis- or trans-resveratrol, or 10 µM SAHA as positive control. After 72 h incubation, 20 µL of assay reagent were added and gently mixed. Measurement of fluorescence was carried out after empirically determined incubation times at 37 °C for each cell line (1.5 h for MiaPaCa; 1.75 h for A498; 2 h for HepG2, Capan-2, HCT-116, HCT-116/p53^(−/−)^, SN12C; 4 h for Hep3B) with a microtiter plate reader (GENios Plus, Tecan, Crailsheim, Germany; recorded fluorescence (550_Ex_/595_Em_), adjusted gain: 60). CTB assay was repeated in three independent experiments, respectively.

### 4.4. Sulforhodamine B Cytotoxicity Assay

Cells were seeded in 24-well plates (2 × 10^4^ cells/well) in a volume of 550 µL as triplicates and incubated overnight. Next, the medium was discarded and replaced with culture medium supplemented with 0.1, 1, 10, or 100 µM cis- or trans-resveratrol, respectively. As positive control, SAHA at 10 µM concentration was used. Complete cell death as control was induced by lysing cells with 1% (*v*/*v*) Triton X-100 (Roth, Karlsruhe, Germany) immediately before SRB staining. Growth inhibition was evaluated after an incubation time of 72 h by SRB assay. During the procedure, medium was discarded and each well was washed once with ice-cold PBS (phosphate buffered saline) and fixed with 10% trichloroacetic acid (TCA) for 30 min at 4 °C. After washing with tap water, cells were dried at 40 °C, then the proteins were stained for 10 min with SRB (0.4% (*w*/*v*) in 1% (*v*/*v*) acetic acid; CAS 3520-42-1, Sigma–Aldrich), and finally, after removing unbound dye by washing with tap water and 1% (*v*/*v*) acetic acid at the end, dried again at 40 °C. Protein-bound dye was resolved with 10 mM Tris base (pH 10.5). After 10 min incubation at room temperature, optical density was measured in triplicates of 80 µL volume per well in 96-well plates with GENios Plus (Tecan; measurement wavelength 550 nm, reference wavelength 620 nm). Data represent the mean of optical density values related to untreated control cells.

### 4.5. Lactate Dehydrogenase Assay

LDH content in the supernatant was determined prior to performing SRB assay. LDH-Mono-P assay (Analyticon, Lichtenfels, Germany) was used, as suggested by the instructor’s manual. Therefore, 10 µL supernatant of each well was mixed with 200 µL of LDH substrate in a 96-well plate and the absorbance measured immediately at 340 nm with microtiter plate reader GENios Plus (Tecan, 5 kinetic cycles, 120 s interval). As positive control, cells were lysed with 1% (*v*/*v*) Triton X-100 (Roth). Each well was measured in triplicates.

### 4.6. Real Time Proliferation Assay

The xCELLigence^®^ SP system (Agilent Technologies Inc., Santa Clara, CA, USA) was used to monitor cell proliferation in real time. HepG2 (1 × 10^4^ cells/well) and Hep3B (2.5 × 10^3^ cells/well) were seeded in a volume of 100 µL in 96-well plates equipped with microelectrode sensors (E-Plate 96, OMNI Life Science GmbH & Co. KG, Bremen, Germany) in which 90 µL of medium had already been submitted for measurement of a zero balance. Treatment was performed by adding 20 µL of medium containing calculated concentrations of the test compounds (see above for final concentrations) to reach a final volume of 210 µL per well. Cell proliferation was recorded by impedance measurement at 30-min intervals over a period of 130 h. Experiments were repeated three times, independently. For data analysis, cell index values were calculated using the Real Time Cell Analyzer (RTCA) Software Pro (2.3.2.) (Agilent Technologies Inc.).

### 4.7. Analysis of Cell Death with TMRE Staining of Mitochondria by Flow Cytometry

Cells (5 × 10^5^) of the different tumor cell lines were seeded in 6-well plates in a volume of 2 mL, and the following day exposed to the resveratrol isomers and SAHA at the above-mentioned concentrations. As control for apoptosis, STS stock solution (Enzo Life Sciences) was added 20 h before flow cytometry at a final concentration of 5 µM. After 72 h of incubation, mitochondrial transmembrane potential damage was determined by TMRE staining. For this purpose, cells were collected after detachment with trypsin, together with medium and PBS used to wash the plates. Cells were centrifuged at 200 g for 4 min, the supernatant discarded, and the cell pellet once washed with PBS. The cell pellet was then resuspended in 100 nM TMRE (Thermo Scientific, Schwerte, Germany) in PBS, and cells were stained for 30 min at 37 °C. Finally, cells were washed again with PBS and resuspended in 1 mL PBS supplemented with 1% (*v*/*v*) FCS. A NovoCyte 2060R flow cytometer (Agilent Technologies Inc.) was used for measurements; data were analyzed with NovoExpress 1.4.1 software (Agilent Technologies Inc.). For each measurement, 1 × 10^4^ events were counted.

### 4.8. Analysis of Cell Cycle with Propidium Iodide Staining by Flow Cytometry

Cells (5 × 10^5^) of the different tumor cell lines were seeded in 6-well plates in a volume of 2 mL, and the following day exposed to the resveratrol isomers and SAHA at the above-mentioned concentrations. As control for apoptosis, STS stock solution (Enzo Life Sciences) was added 20 h before flow cytometry at a final concentration of 5 µM. After 72 h of incubation, cell cycle analysis was determined by PI staining. For this purpose, cells were collected after detachment with trypsin, together with medium and PBS used to wash the plates. Samples were centrifuged at 200 g for 4 min, the supernatant discarded, and the cell pellet washed once with cold PBS (4 °C). Subsequently, the cells were fixated with 1 mL ice cold 75% ethanol and mixed gently, and then placed in the fridge for at least 2 h. Thereafter, 2 mL of cold PBS was added and samples were centrifuged again (200 g, 10 min, 4 °C), followed by carefully aspirating the supernatant. Cells were then washed once with cold PBS and stained with PI/RNAse solution while gently vortexing (PI 50 µg/mL (Sigma–Aldrich) + 100 µg/mL RNAse (Sigma–Aldrich)), and then incubated for at least 20 min at 4 °C in the dark. A NovoCyte 2060R flow cytometer (Agilent Technologies Inc.) was used for measurements; data were analyzed with NovoExpress 1.4.1 software (Agilent Technologies Inc.). For each measurement, 1 × 10^4^ events were counted.

### 4.9. ApoTox-Glo^TM^ Triplex Assay

The ApoTox-Glo^TM^ Triplex Assay (Promega) measures cell viability by determining protease activity in living cells and uses a specific fluorogenic cell-permeable peptide substrate (glycyl-phenylalanyl-aminofluorocoumarin; GF-AFC). The assay was performed according to manufacturer’s instructions. In brief, 1 × 10^4^ cells of the different tumor cell lines were seeded in 96-well assay plates and grown for 24 h. Subsequentially, cells were exposed for 24 h to the resveratrol isomers, SAHA, and STS (Enzo Life Sciences) as a control for apoptosis at the above-mentioned concentrations in a total volume of 100 µL. Finally, 20 µL of Viability/Cytotoxicity reagent with GF-AFC substrate and bis-alanylalanyl-phenylalanyl-rhodamine 110 (bis-AAF-R110) substrate was added to all wells. After 30-40 min incubation at 37 °C, fluorescence was measured at 400_Ex_/505_Em_ with the Synergy H1 multiplate reader (BioTek) to determine cell viability.

## 5. Conclusions

In the present study, both the trans-isomer and the cis-isomer were shown to have antiproliferative activity against hepatoma, colon carcinoma, pancreatic carcinoma, and renal carcinoma cell lines. Except for the Capan-2 cell line, trans-resveratrol was regularly found to be more effective than cis-resveratrol, although the latter also showed pronounced activity against tumor cells itself. However, the effects found were predominantly independent of p53 status, except in the pancreatic carcinoma cell lines, where the p53 mutant was significantly more sensitive than the p53 wild type. Based on other studies showing that the two resveratrol isomers even have opposing effects with respect to PARP1 activation, and the finding that methoxylated cis-derivatives are superior to the corresponding trans-derivatives in their antitumor efficacy, it becomes clear that not only trans-resveratrol but also cis-resveratrol represents an extremely interesting lead structure for the development of drugs for the prevention and therapy of tumor diseases with particularly unfavorable prognosis.

## Figures and Tables

**Table 1:** Tumor entities and p53 status of tumor cell lines employed throughout this study.

Tumor Entity	Cell Line	p53 Status	Reference
Hepatoma	HepG2	wt	[31]
	Hep3B	partial deletion, absence of transcripts/protein	[32]
Colon carcinoma	HCT-116	wt	[33]
	HCT-116/p53^(−/−)^	deletion of both p53 alleles	[34]
Pancreatic cancer	Capan-2	wt	[35]
	MiaPaCa-2	missense mutation (exon 7), Arg > Trp	[31]
Renal cell carcinoma	A498	wt	[36]
	SN12C	nonsense mutation (exon 10), Glu > Stop	[37]

## Data Availability

Not applicable.

## Supplementary Materials

The following are available online, Figure S1: Western Blot analysis of p53 protein, Figure S2: Reduced viability of pancreas- and kidney carcinoma cells with different p53 status by trans- and cis-resveratrol shown by CTB assay, Figure S3: Reduced cell mass of pancreas- and kidney carcinoma cells with different p53 status by trans- and cis-resveratrol shown by SRB assay, Figure S4: LDH release of hepatocellular, colorectal, pancreas- and kidney carcinoma cells with different p53 status by trans- and cis-resveratrol shown by LDH assay, Figure S5: TMRE staining of mitochondria in hepatocellular and colorectal carcinoma cells with different p53 status after treatment with trans- and cis-resveratrol evaluated by flow cytometry, Figure S6: Viability assay of hepatocellular and colorectal carcinoma cells with different p53 status after treatment with trans- and cis-resveratrol.

## Abbreviations

ATCC	American Type Culture Collection
bis-AAF-R110	bis-alanylalanyl-phenylalanyl-rhodamine 110
CDK	cyclin-dependent kinase
COX-2	cyclooxygenase-2
CTB	CellTiter-Blue^®^
DMEM	Dulbecco´s modified Eagle´s medium
DMSO	dimethyl sulfoxide
DSMZ	Deutsche Sammlung von Mikroorganismen und Zellkulturen
ECACC	European Collection of Authenticated Cell Cultures
ERK1/2	extracellular signal-regulated kinase 1/2
FCS	fetal calf serum
GF-AFC	glycyl-phenylalanyl-aminofluorocoumarin
HCC	hepatocellular carcinoma
HRP	horseradish peroxidase
LDH	lactate dehydrogenase
MAPK	mitogen-activated protein kinase
NKG2D	natural-killer group 2, member D
NOS-2	nitric oxidase synthase-2
OD	optical density
PARP	poly-ADP-ribose polymerase
PBS	phosphate buffered saline
PGE2	prostaglandin E2
PI	propidium iodide
PKC	protein kinase C
PTK	protein-tyrosine kinase
PVDF	polyvinylidene difluoride
ROS	reactive oxygen species
RTCA	Real Time Cell Analyzer
SAHA	suberoylanilide hydroxamic acid
SDS	sodium dodecyl sulfate
SRB	sulforhodamine B
STS	staurosporine
TBS	Tris-buffered saline
TBST	TBS-Tween 20
TCA	trichloroacetic acid
TMRE	tetramethylrhodamine ethyl ester
