# Supplementary material for: Comparative Analysis of the Antitumor Activity of Cis- and Trans-Resveratrol in Human Cancer Cells with Different p53 Status
Source: Molecules. 2021 Sep 14;26(18):5586. doi: 10.3390/molecules26185586 (PMC8466563; doi:10.3390/molecules26185586)
Supplement: Supplementary file 1 [file molecules-26-05586-s001.zip › molecules-1321474-supplementary.pdf]

“Comparative analysis of the antitumor activitiy of cis- and trans-resveratrol in human cancer cells with different p53 status”

Supplementary figures

Figure S1

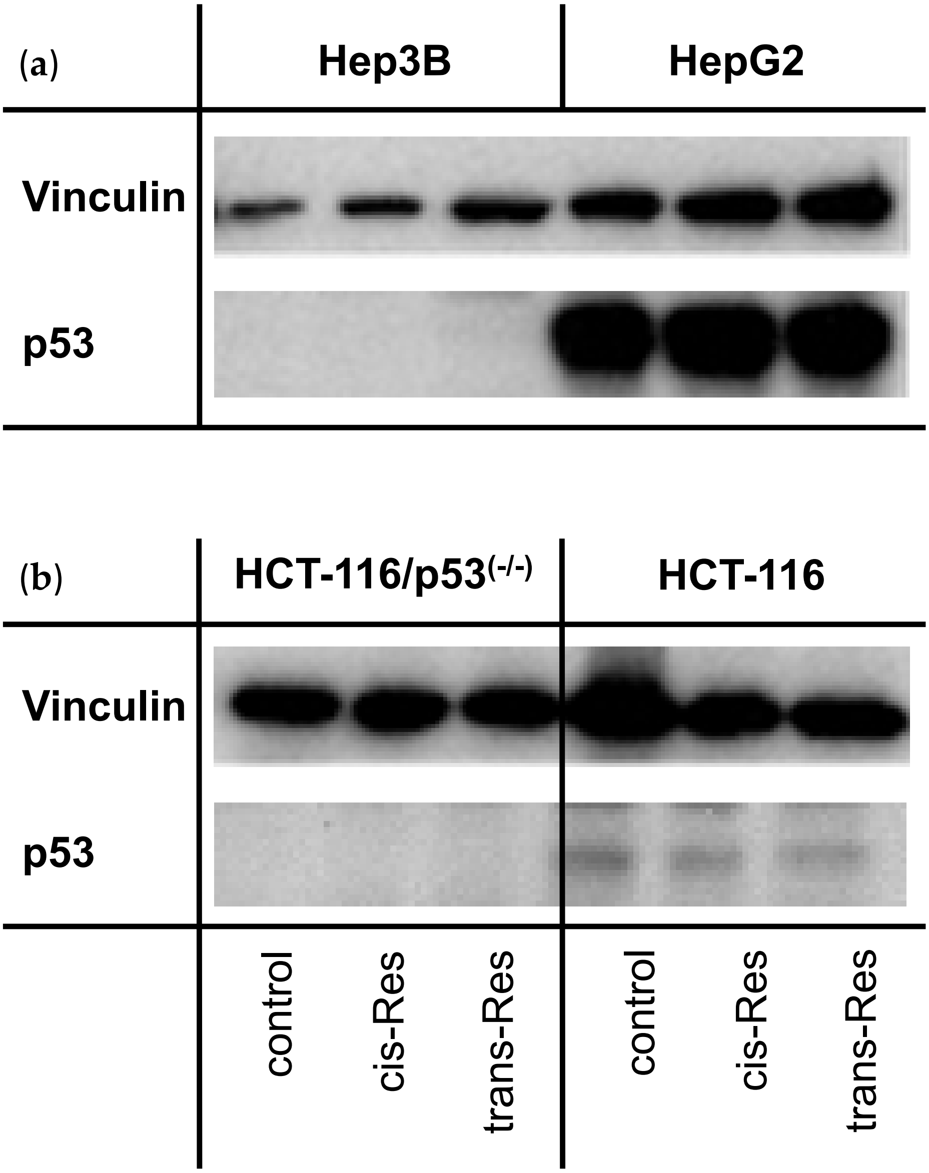

Figure S1. Western Blot analysis of p53 protein in (**a**) Hep3B, HepG2, and (**b**) HCT-116/p53$^{(-/-)}$ and HCT-116 cells 72 h after exposure to 10 μM cis- or trans-resveratrol. Vinculin as housekeeping protein served as control for equal protein loading. Res, resveratrol.

Figure S2

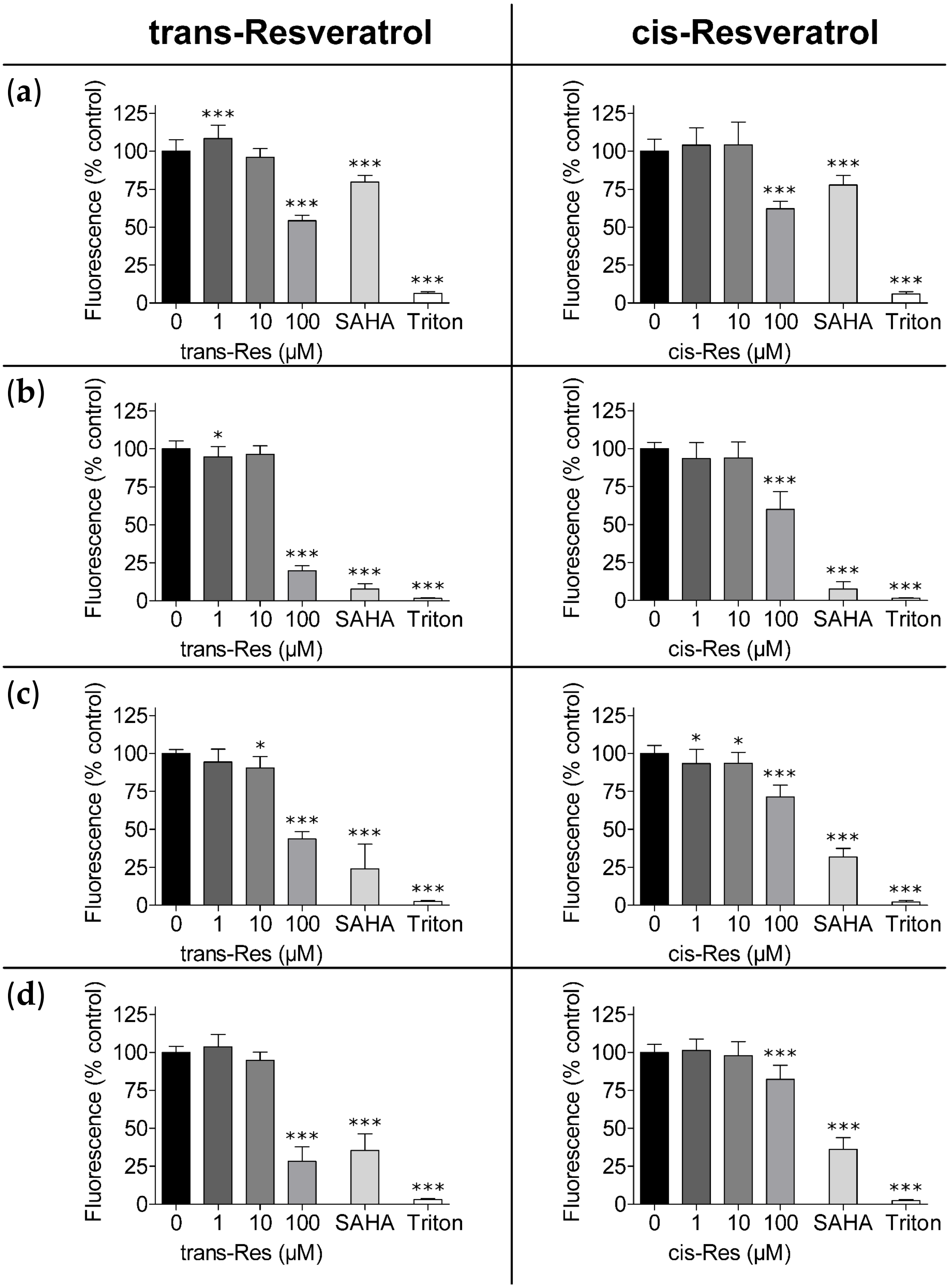

Figure S2. Reduced viability of pancreas- and kidney carcinoma cells with different p53 status by trans- and cis-resveratrol shown by CTB assay. (**a**) Capan-2, (**b**) MiaPaCa-2 (pancreas carcinoma), (**c**) A498, and (**d**) SN12C (kidney carcinoma) cells were exposed to different concentrations of trans- and cis-resveratrol (1 µM, 10 µM, 100 µM) for 72 h. 10 µM SAHA served as positive control and 1% (v/v) Triton X-100 as a cell death control. The experiments were replicated in three independent experiments, each performed in pentaplicates. Results are shown as percentages compared to the untreated control. Error bars represent mean ± SD, statistical analysis with the Dunnet's multiple comparison test, confidence interval 95%. *: $p \leq 0.05$; **: $p \leq 0.01$; ***: $p \leq 0.001$. CTB, CellTiter Blue®; Res, resveratrol; SAHA, suberoylanilide hydroxamic acid.

Figure S3

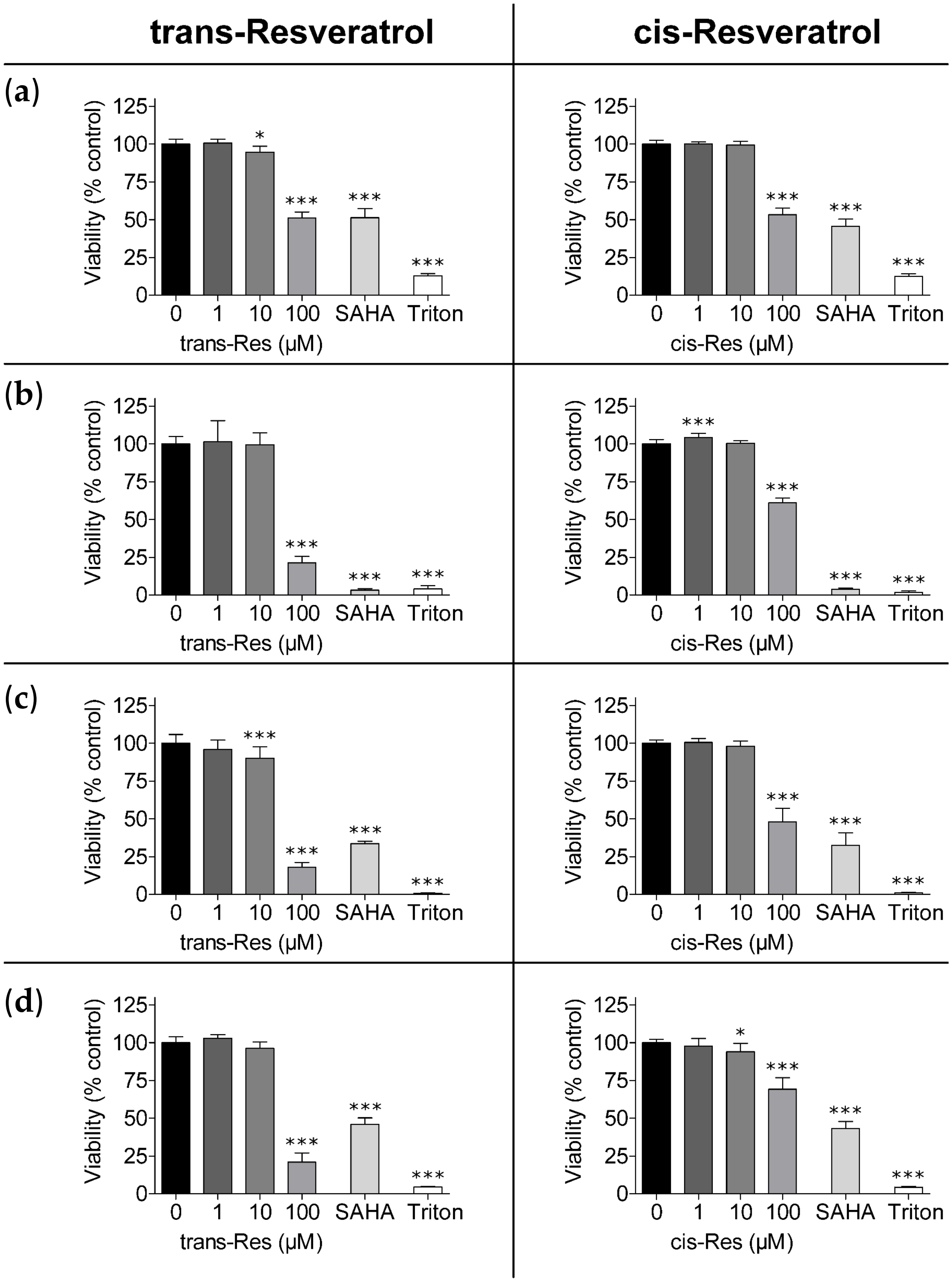

Figure S3. Reduced cell mass of pancreas- and kidney carcinoma cells with different p53 status by trans- and cis-resveratrol shown by SRB assay. (**a**) Capan-2, (**b**) MiaPaCa-2 (pancreas carcinoma), (**c**) A498, and (**d**) SN12C (kidney carcinoma) cells were exposed to different concentrations of trans- and cis-resveratrol (1 µM, 10 µM, 100 µM) for 72 h. 10 µM SAHA served as positive control and 1% (v/v) Triton X-100 as cell death control. The experiments were replicated in three independent experiments, each performed in triplicates. Results are shown as percentages compared to the untreated control. Error bars represent mean ± SD, statistical analysis with the Dunnet's multiple comparison test, confidence interval 95%. *: $p \leq 0.05$; **: $p \leq 0.01$; ***: $p \leq 0.001$. Res, resveratrol; SAHA, suberoylanilide hydroxamic acid; SRB, sulforhodamine B.

Figure S4

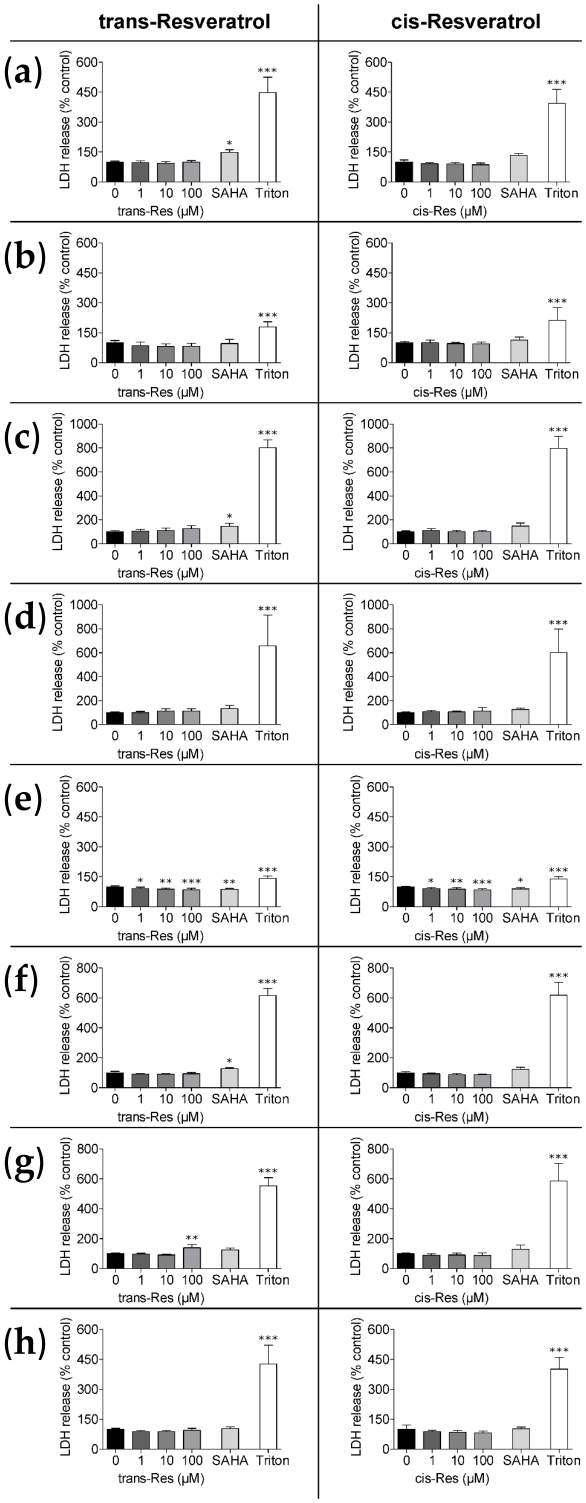

Figure S4. LDH release of hepatocellular, colorectal, pancreas- and kidney carcinoma cells with different p53 status by trans- and cis-resveratrol shown by LDH assay. (**a**) HepG2, (**b**) Hep3B (hepatoma), (**c**) HCT-116, (**d**) HCT-116/p53$^{(-/-)}$ (colon carcinoma), (**e**) Capan-2, (**f**) MiaPaCa-2 (pancreas carcinoma), (**g**) A498, and (**h**) SN12C (kidney carcinoma) cells were exposed to different concentrations of trans- and cis-resveratrol (1 µM, 10 µM, 100 µM) for 72 h. 10 µM SAHA served as positive control and 1% (v/v) Triton X-100 as cell death control. The experiments were replicated in three independent experiments, each performed in triplicates. Results are shown as percentages compared to the untreated control. Error bars represent mean ± SD, statistical analysis with the Dunnet's multiple comparison test, confidence interval 95%. *: $p \leq 0.05$; **: $p \leq 0.01$; ***: $p \leq 0.001$. Res, resveratrol; SAHA, suberoylanilide hydroxamic acid; LDH, lactate dehydrogenase.

Figure S5

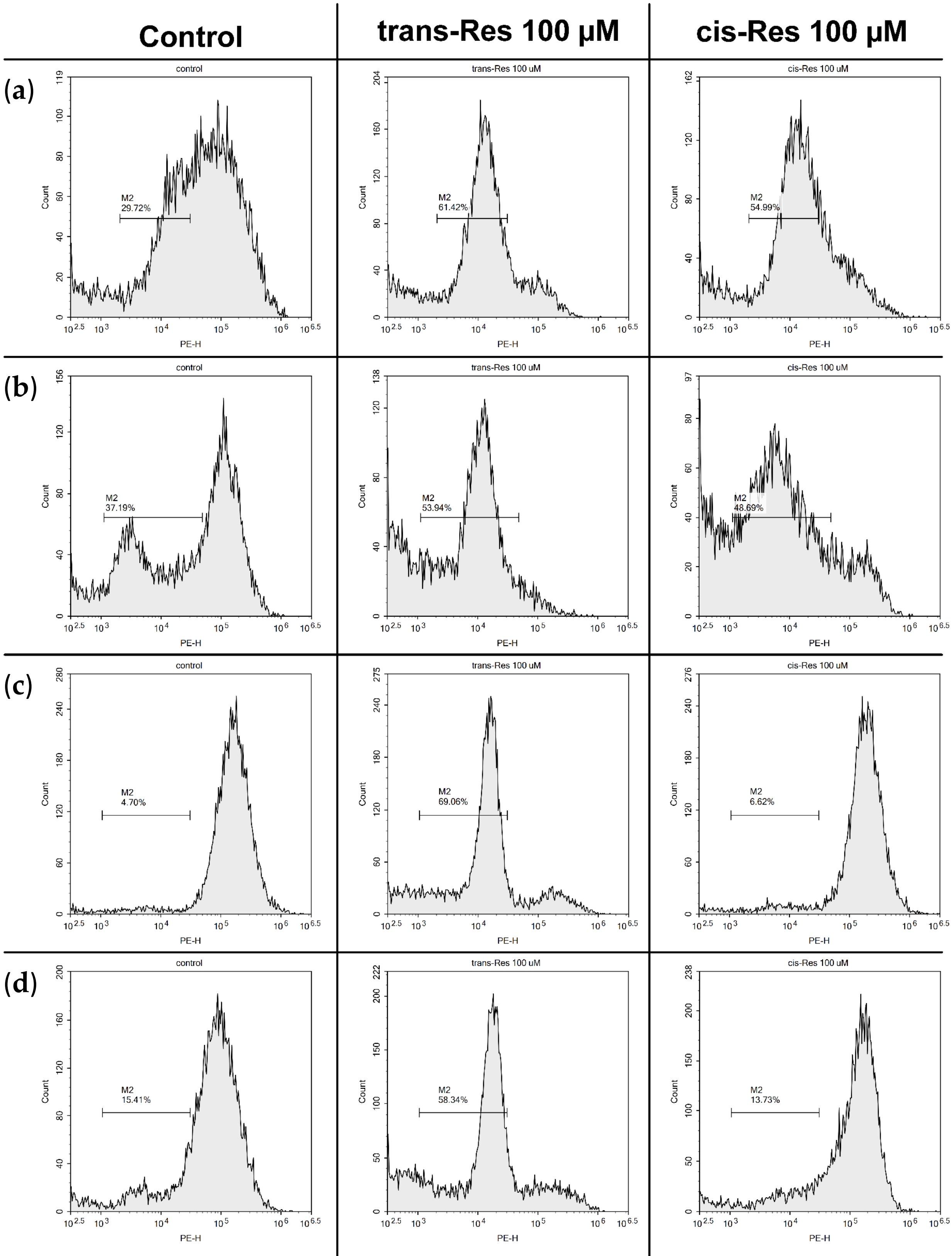

Figure S5. TMRE staining of mitochondria in hepatocellular and colorectal carcinoma cells with different p53 status after treatment with trans- and cis-resveratrol evaluated by flow cytometry. (**a**) HepG2, (**b**) Hep3B (hepatoma), (**c**) HCT-116, and (**d**) HCT-116/p53$^{(-/-)}$ (colon carcinoma) cells were exposed to trans- and cis-resveratrol in a concentration of 100 µM for 72 h. Shown are exemplary histograms of one out of three independent experiments. Res, resveratrol; TMRE, tetramethylrhodamine ethyl ester.

Figure S6

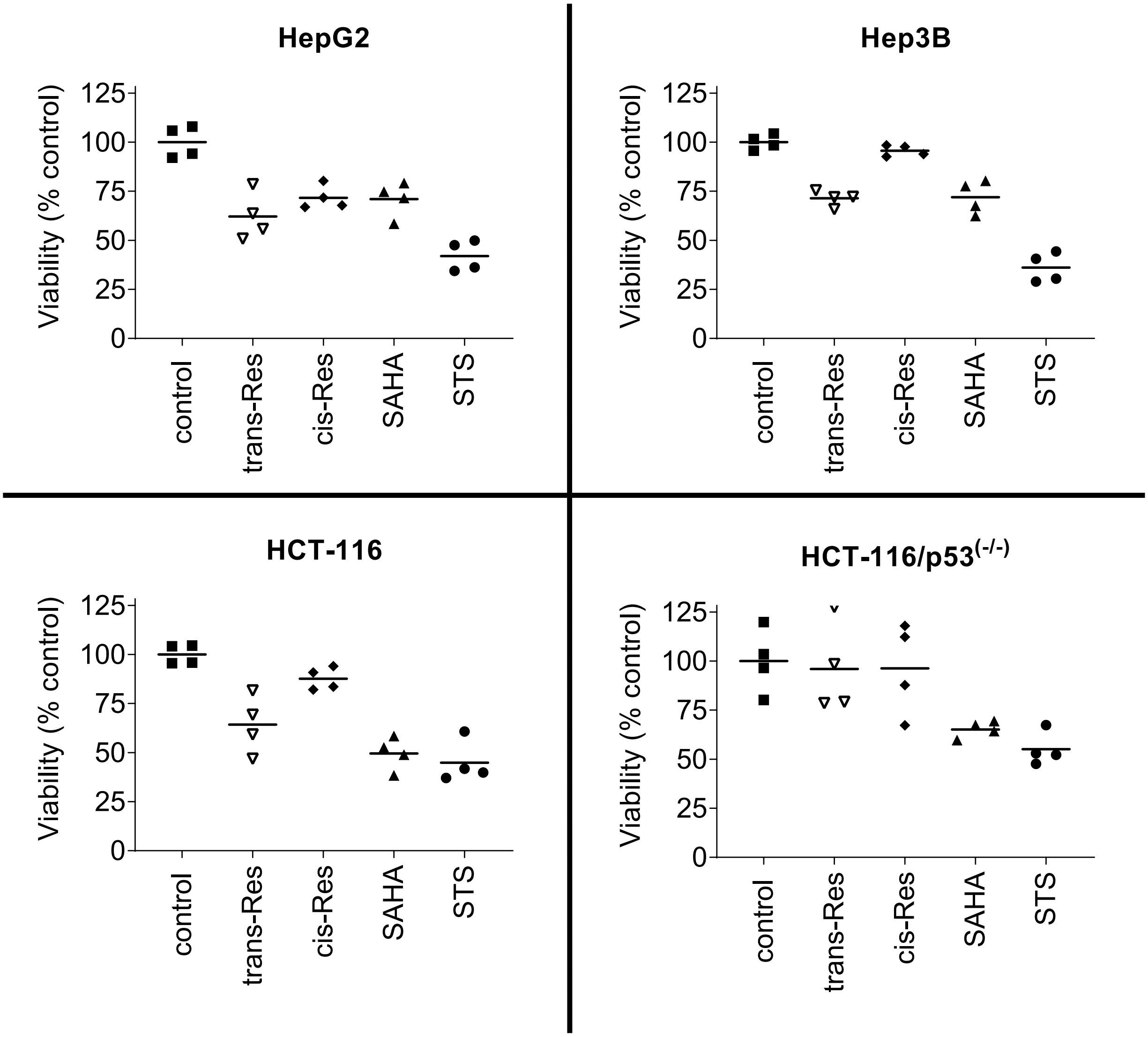

Figure S6. Viability assay of hepatocellular and colorectal carcinoma cells with different p53 status after treatment with trans- and cis-resveratrol. HepG2, Hep3B (hepatoma), HCT-116, and HCT-116/p53$^{(-/-)}$ (colon carcinoma) cells were exposed to trans- and cis-resveratrol in a concentration of 100 μM for 24 h. 10 μM SAHA served as positive control and 5 μM STS as control for apoptosis induction. Shown are the mean of two independent experiments, each performed in duplicates. The symbols correspond to individual experimental measurements, which are shown as scatter dot plot. Results are presented as percentages compared to the untreated control. Res, resveratrol; SAHA, suberoylanilide hydroxamic acid; STS, staurosporine.
